# Response letter

**DOI:** 10.1177/11297298231218663

**Published:** 2024-01-02

**Authors:** Letty V van Vliet, Tammo Delhaas, Barend ME Mees, Maarten G Snoeijs

**Affiliations:** 1 Department of Biomedical Engineering, CARIM School for Cardiovascular Diseases, Maastricht University, Maastricht, The Netherlands; 2 Department of Vascular Surgery, Maastricht University Medical Centre, Maastricht, The Netherlands

We express our gratitude to Yadav and Scheltinga for their interest in and response to our article on the relation between guideline recommendations on minimal blood vessel diameters and arteriovenous fistula outcomes.^1^

In our study, we found that upper arm arteriovenous fistulas with preoperative blood vessel diameters <3 mm had similar vascular access function as fistulas created with larger blood vessels, whereas forearm arteriovenous fistulas with preoperative blood vessel diameters <2 mm had poor clinical outcomes.^1^ In line with our findings, Yadav et al.^2^ found that the venous diameter cut-off recommended by the clinical guidelines did not reliably predict vascular access patency. Despite the association of blood vessel diameters with fistula maturation, establishing a strict cut-off value that consistently leads to successful vascular access outcomes remains challenging.^3^

**Figure 1.**
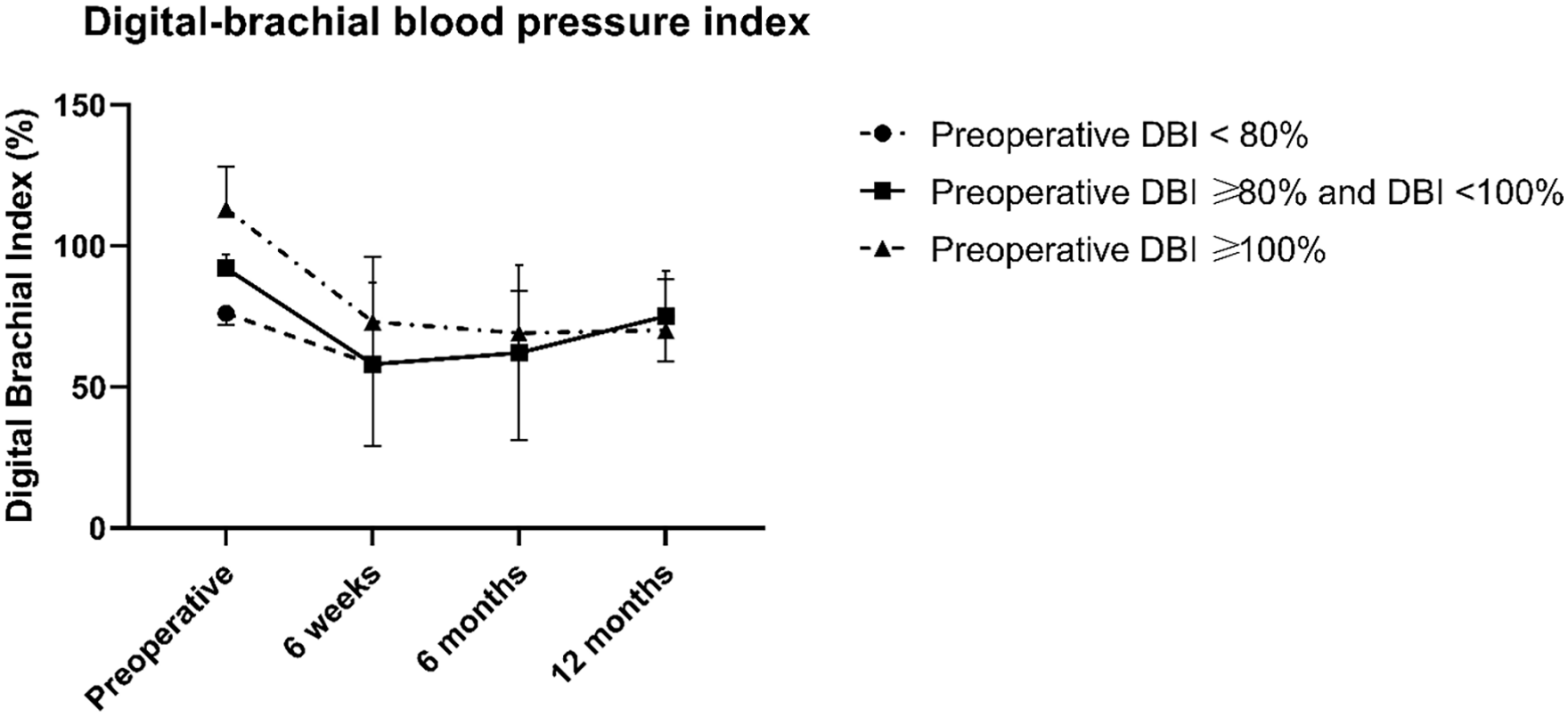
Digital-brachial blood pressure index. Mean digital-brachial blood pressure index (DBI) with standard deviation over time according to preoperative DBI groups.

Therefore, Yadav and Scheltinga as well as our study group emphasize the importance of considering the quality of blood vessels beyond their diameters in vascular access decision-making. Yadav and Scheltinga have presented the digital-brachial blood pressure index (DBI) as an indicator of blood vessel quality that was associated with vascular access function in their retrospective patient cohort.^2^ We sought to replicate their findings in the multicenter prospective cohort of the Shunt Simulation Study.^4^ Of 166 patients with preoperatively measured DBI, 83 had a normal DBI (⩾80% and <100%), 68 had a high DBI (⩾100%), and 15 had a low DBI (<80%). Patient characteristics did not differ significantly between these groups, although patients with a low DBI tended to have diabetes more frequently (Table 1). Despite the initial differences in preoperative DBI, postoperative DBI was comparable within the first year after fistula creation (Figure 1). Parameters of vascular access function such as fistula maturation assessed by duplex ultrasonography, development into a functional vascular access, access-related intervention rates, and primary and secondary patency were similar between groups with low, normal, and high preoperative DBI (Table 1).

**Table 1.** Patient characteristics and clinical outcomes according to digital-brachial blood pressure index.

	DBI <80% *N* = 15	DBI ⩾80% and DBI <100% *N* = 83	DBI ⩾100% *N* = 68	*p*
*Patient characteristics*				
Age (years)	67 (12)	66 (12)	66 (12)	0.96
Sex (male)	67%	57%	69%	0.27
BMI (kg/m^2^)	30 (7)	28 (5)	27 (5)	0.27
Diabetes	67%	46%	35%	0.07
Hypertension	100%	89%	85%	0.26
Duplex measurements				
Arterial diameter (mm)	2.8 (0.7)	2.8 (0.8)	3.1 (1.0)	0.22
Venous diameter (mm)	2.8 (0.6)	2.6 (0.8)	2.7 (0.9)	0.62
Preoperative systolic blood pressure				
Digital artery (mmHg)	123 (19)	153 (26)	161 (26)	<0.01
Brachial artery (mmHg)	163 (22)	166 (26)	144 (23)	<0.01
Clinical outcomes				
Maturation at 6 weeks*	53%	67%	71%	0.41
Functional vascular access**	64%	69%	73%	0.83
Access-related intervention rate (/patient-year)	1.70	1.69	1.58	0.61
Patency at 1 year***				
Primary patency	50%	41%	41%	0.89
Secondary patency	70%	80%	83%	0.58

* Maturation was defined as brachial artery flow ⩾500 mL/min and venous diameter of ⩾4 mm on duplex ultrasound at 6 weeks after surgery.

** Functional vascular access was defined as cannulation with two needles at the prescribed access circuit flow for at least 6 hemodialysis sessions in 30 days within 4 months after surgery (for patients on dialysis at the time of vascular access creation or who started dialysis within 4 months after vascular access creation), and as starting dialysis with the index arteriovenous fistula (for patients who had not yet started dialysis treatment at 4 months after vascular access creation).

*** Patencies were compared between groups with log rank tests.

In contrast to the findings of Yadav and Scheltinga, our analysis showed that patients with abnormal DBI did not have an increased risk of vascular access failure. Yadav and Scheltinga hypothesize that low DBI reflects the presence of atherosclerosis and that high DBI indicates increased vascular stiffness and decreased vascular compliance. However, we must acknowledge certain limitations to the DBI as a general indicator of blood vessel quality. As the DBI is the relative systolic pressure difference between the brachial and digital arteries, it is unable to provide insight into the quality of the specific artery used for the arteriovenous fistula. Undeniably, none of the arteries distal to the brachial artery are involved when creating upper arm fistulas. Additionally, DBI does not account for the quality of veins that play a pivotal role in fistula maturation and stenosis development.^5^

In summary, the post hoc analysis of the prospective, multicenter Shunt Simulation Study shows that arteriovenous fistula outcomes were not associated with preoperative DBI. We encourage further exploration of methods to evaluate blood vessel quality before arteriovenous fistula creation, with the ultimate aim to improve vascular access outcomes.
